# Exploring the nexus between financial inclusion, governance, and carbon emissions in SAARC countries

**DOI:** 10.1016/j.heliyon.2024.e40985

**Published:** 2024-12-06

**Authors:** Jafir Mehmood, Yang Jinghan, Jing Wang, Maqsood Ahmad

**Affiliations:** a College of Economics and Management, Northwest A&F University, Yangling, Shaanxi, 712100, PR China; b School of Economics and Management, Tarim University, Alar, Xinjiang, 843300, PR China; c School of Accounting and Finance, The Hong Kong Polytechnic University, Hong Kong

**Keywords:** Institutional quality, Financial inclusion, Carbon emissions, GMM approach, SAARC

## Abstract

This study examines the impact of financial inclusion (FI) and institutional quality (INSQ) on carbon dioxide (CO_2_) emissions in South Asian Association for Regional Cooperation (SAARC) economies, using data from 2004 to 2022. The hypotheses were tested using a generalized method of moments (GMM) approach. Beside, a robust moment method quantile regression (MM-QR) static model and Granger causality tests were employed to validate the results. The findings indicate that FI indicators, such as bank branches of commercial banks (BOCB) and automated teller machines (ATMs) are positively associated with CO_2_ emissions. Similarly, the INSQ has a significant positive impact on CO_2_ emissions. Control variables, including foreign direct investment (FDI), financial development (FD), and population growth (PG), are also positively linked to CO_2_ emissions, whereas globalization (GI) has a negative impact. Robustness tests confirm that the effects of FI and INSQ on CO_2_ emissions vary across economic contexts, with unidirectional causality observed between BOCB and CO_2_ and bidirectional causality between ATMs and CO_2_. This study highlights the need for policymakers in SAARC countries to balance their economic development and environmental sustainability. Integrating environmentally friendly technologies and practices into financial and institutional development strategies is essential. Promoting green banking, strengthening environmental regulations, and leveraging globalization for cleaner technologies can help mitigate the adverse effects of FI and INSQ on CO_2_ emissions. This study underscores the importance of incorporating environmental considerations into economic and financial policies to achieve sustainable development.

## Highlights

• This study found a positive association between financial inclusion, institutional quality, and CO_2_ emissions.
• There is long-term relationship between financial inclusion, institutional quality, and carbon emissions in SAARC countries.
• To explore the heterogenous impacts of financial inclusion on carbon emissions in SAARC countries using GMM and robust MM-QR approach.
• SAARC countries must promote financial inclusion while incorporating environmentally friendly technological breakthroughs and institutional quality regulations into their climate change practices.

## 1 Introduction

The preservation of environmental sustainability (EMS) has become a top priority in the pursuit of sustainable development, particularly in response to pressing global challenges such as climate change and ecological degradation [[1], [2], [3]]. Consequently, there is increasing interest among academics, policymakers, and practitioners in understanding the diverse factors that influence CO_2_ emissions. Among these factors, FI and INSQ have emerged as crucial determinants that influence both economic growth and environmental sustainability, especially in developing regions such as South Asia. The INSQ, which reflects the strength of regulatory frameworks, governance structures, and enforcement of laws, plays a crucial role in shaping economic outcomes and environmental policies. However, the relationship between INSQ and EMS remains contested. In theory, robust institutions can implement policies that promote EMS and reduce carbon emissions. However, empirical evidence suggests that institutional improvements may, in certain contexts, coincide with increased economic activity and greater environmental degradation.

Similarly, FI broadens access to financial services, acting as a catalyst for economic development by facilitating investment, consumption, and entrepreneurial activities. However, its environmental implications are mixed, with some studies linking FI to higher energy consumption and CO_2_ emissions, while others highlight its potential to promote sustainable development. Recent research highlights the role of the well-established financial sector in supporting climate-change mitigation efforts [4]. A robust financial system not only promotes economic growth but also encourages the adoption of eco-friendly technologies and practices, contributing to lower CO_2_ emissions [5]. Financial institutions are vital in improving environmental quality because well-developed financial markets incentivize firms to adopt cleaner technologies, reduce pollution, and enhance ecological performance [6].

FI is recognized as a vital driver of economic growth, enabling individuals and enterprises to manage their finances and participate in economic activities [7]. According to the World Bank, FI encompasses a range of financial services, including transactions, credit, loans, and investments. While FI fosters economic growth and poverty reduction, developing economies, especially those in the SAARC region, face the dual challenges of achieving FD and environmental sustainability. These nations are experiencing rapid increases in energy consumption, leading to substantial CO_2_ emissions and ecological degradation [8].

Rapid economic growth in South Asia has led to many environmental challenges like deforestation, weak institutions, and increasing the agricultural greenhouse gas emissions [9,10]. Limited financial services and institutional support further exacerbate these issues, affecting over a billion people in the region [11]. Implementing green technologies like solar energy could substantially reduce carbon emissions, but capital and institutional barriers impede advancement [12]. However, FD has the potential to drive the adoption of green technologies, enhance energy efficiency, and reduce emissions [13]. Therefore, it is essential to focus on FI systems and institutional governance when addressing economic growth and environmental protection.

**Fig. 1:**
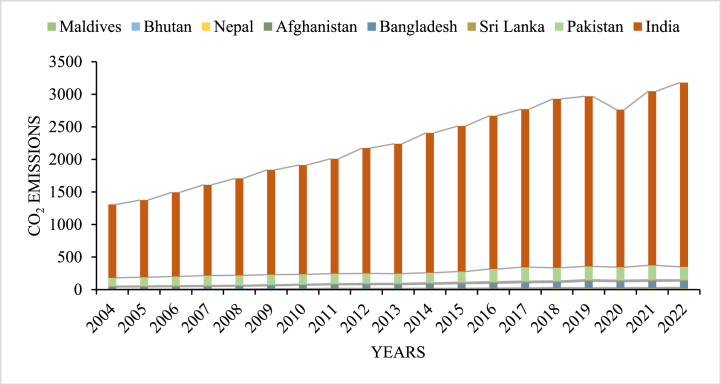
Territorial emissions in MtCO₂ from 2004 to 2022, Source: Author calcultion.

Promoting EMS in the SAARC region requires financial services that reach socioeconomically disadvantaged populations and support strong institutional frameworks. The Asian Development Bank identifies South Asia as a highly vulnerable region to climate change [14]. With eight SAARC economies representing 25 % of the global population, this region is a significant contributor to increasing CO_2_ emissions (see Fig. 1). Understanding how FI and INSQ influence environmental outcomes is crucial for promoting sustainable development in these countries. The SAARC countries, including Pakistan, Sri Lanka, Bangladesh, Afghanistan, Nepal, Maldives, Bhutan, and India, provide a unique context to explore the relationship between FI, INSQ, and CO_2_ emissions. These economies are characterized by rapid economic growth, expanding financial infrastructure, and diverse institutional capacities, along with significant environmental challenges. The dual goals of fostering economic development and addressing EMS are particularly relevant for SAARC nations, where the need for financial inclusiveness and improved governance must be balanced with the imperative of reducing carbon emissions and mitigating climate change.

Despite extensive research on FI, INSQ, and environmental sustainability, their combined effects on CO_2_ emissions in the SAARC region remain unexplored [[15], [16], [17], [18], [19], [20], [21]]. To the best of our knowledge, no prior study has comprehensively examined these relationships using a GMM approach with MM-QR and Granger causality tests. Moreover, prior studies have produced mixed findings, with some indicating that FI and INSQ contribute to environmental degradation and others arguing that these factors can help mitigate emissions under certain conditions. Addressing these mixed findings is crucial because inconsistent results create uncertainty for policymakers and stakeholders who rely on evidence-based strategies to combat climate change.

This study bridges these gaps by examining the impact of FI and INSQ on CO_2_ emissions in SAARC economies from 2004 to 2022, utilizing a dynamic GMM approach with MM-QR and Granger causality tests to provide dynamic insights. Furthermore, while previous research has explored FI and EMS in various contexts, using diverse indices and methods for the desired results [17,18,22], this study addresses the gap by investigating the heterogeneous effects of two key proxies of FI—BOCB and ATMs—on CO_2_ emissions in the SAARC countries. Despite their importance in promoting FI and improving INSQ, the effects of these variables on carbon emissions in the SAARC region have not been explored separately. This research also contributes to the literature on achieving “Sustainable Development Goals” (SDGs), particularly SDG 8 (decent work and economic growth) and SDG 9 (industry, innovation, and infrastructure) [23,24]. By expanding production and providing dynamic financial services, SAARC countries can pursue a path toward achieving the SDGs by 2030. Overall, this study contributes to the literature by highlighting the importance of FI and INSQ in achieving EMS. The results obtained from our GMM model and quantile analysis provide valuable insights for policymakers and stakeholders in SAARC nations, facilitating them to devise targeted strategies to enhance environmental sustainability.

The remaining part of this paper is organized as follows. Section 2 summarizes the previous literature regarding the association between FI and INSQ with CO_2_ and develops the hypotheses of our study. Section 3 explains the data collection process and methodology. Section 4 presents the empirical findings and a discussion. Section 5 provides conclusions and discusses the practical implications of this study.

## 2 Literature review and hypotheses development

Several researchers have investigated the several factors that influence carbon emissions to achieve the environmental sustainability [[25], [26], [27], [28], [29]]. For instance Ref. [30], highlighted the positive environmental impacts achieved through multiple methods such as providing loans at reduced rates, integrating green technologies, and intensifying climate change and conservation efforts [17]. An efficient financial system has the potential to mitigate climate change and facilitate the development of a green environment [15,18,31,32]. However [33], argued that financial growth might exacerbate environmental degradation because of excessive industrial production and resource utilization. On the other hand [34], examined the negative impact of these factors on ecological degradation, while [30] demonstrated that FI contributed to improving EMS.

In addition to financial factors, INSQ has emerged as a critical determinant of environmental outcomes. Strong institutions with sound governance frameworks can help enforce environmental regulations and promote sustainable practices [35]. Ref. [36] emphasized that better INSQ facilitates the implementation of eco-friendly policies, reducing CO_2_ emissions. Conversely, weak institutional frameworks can hinder environmental progress by allowing unsustainable industrial practices to continue [37]. While improvements in INSQ can support environmental sustainability, they must be paired with robust policy measures to mitigate any potential negative impacts on the environment.

### 2.1 Hypotheses development

#### 2.1.1 Financial inclusion and carbon emissions

Previous studies have explored the relationship between FI and CO_2_ emissions, yielding mixed empirical evidence. Ref. [7] who examined 31 Asian nations, and [19], who analyzed 23 OECD member states, both concluded that expanding FI correlates with increased carbon emissions. Similarly [35], identified an inverted U-shaped relationship between FI and climate change across 103 countries, consistent with the environmental Kuznets curve paradigm. However, research on this phenomenon remains limited and inconsistent.

For instance [38], investigated the relationship between FI, infrastructure development, and environmental impact in OECD countries, concluding that while FI exacerbates environmental degradation, infrastructure development negatively affects environmental quality (EQ). Ref. [39] also explored the dynamics of FI, CO_2_ emissions, and national governance across 65 countries, highlighting the moderating role of governance in reducing FI's adverse environmental effects. On the other hand [40], provided evidence that expanding FI could help mitigate its environmental consequences. Ref. [41] examined the link between FI and ecological footprints in 24 OIC member nations, finding a strong positive correlation with environmental degradation. Conversely [18], argued that FI enhances EQ, particularly in emerging economies where a negative association with CO_2_ emissions was observed. Ref. [34] supported this negative impact of FI on carbon emissions in 15 pollution-intensive countries. The study by Ref. [21], which utilized the STIRPAT framework to analyze data from 102 countries from 2004 to 2020, adds depth to this discussion. Their findings indicated an N-shaped relationship between FI and carbon emissions, transitioning from an inverted U-shape to a U-shape as the FI increased. This nonlinear pattern is more pronounced in developing countries than it is in advanced economies. The present study differs methodologically from that of [21], which utilized a composite index to measure FI derived from five FI proxies via principal component analysis, this study employed two main FI indicators with INSQ, BOCB and ATMs, using the latest frequency material and methods. Furthermore, the separate effects of these two indicators on carbon emissions are investigated. This distinction in FI measurements may lead to different interpretations of how FI influences carbon emissions in different contexts.

The literature presents a complex understanding of the FI-CO_2_ nexus. While some studies suggest that increased FI leads to higher CO_2_ emissions and ecological degradation, others argue that improved governance and contextual factors, such as financial systems in developed and emerging economies, can mitigate environmental harm. Based on the prior literature, the following relationship is proposed:

H1 FI significantly influences CO_2_ emissions in SAARC economies.

#### 2.1.2 Institutional quality and carbon emissions

Scholars have also studied the relationship between INSQ and CO_2_ emissions, with inconsistent findings. Some argue that features of INSQ, such as democracy and corruption, can reduce carbon emissions, while others suggest that these features can lead to increased CO_2_ emissions. Ref. [42] found an adverse link between INSQ and CO_2_ emissions in South Asian countries. Similarly [43], concluded that INSQ indirectly and negatively affected EQ. Ref. [44] argue that poor governance and institutions contribute to environmental degradation. Conversely [45], demonstrated that domestic institutes promote economic growth and reduce CO_2_ emissions, emphasizing the significance of trade openness, INSQ, and energy consumption in economic development. Ref. [46] examined in BRICS economies, recommended investing in eco-friendly technologies and improving INSQ for sustainable environmental improvement.

The literature reveals a complex relationship between EQ and institutional factors. While some studies suggest that certain INSQ features may mitigate carbon emissions, others indicate potential adverse effects. The consensus emphasizes the importance of investing in eco-friendly technologies and improving INSQ for sustainable ecological progress. Building on this discourse, the following hypothesis is proposed:

H2 INSQ has a significant influence on CO_2_ emissions in SAARC economies.

To facilitate broader cross-national and cross-regional evaluations, Table 1 provides a comprehensive overview of studies investigating the relationships between FI, INSQ, and carbon emissions. This table serves as a summary of the key findings across various contexts, highlighting the diverse methodologies and outcomes of prior research. The inclusion of both developed and emerging economies ensures a balanced perspective on how these factors interact across governance frameworks and financial systems.

**Table 1:** Overview of related literature published.

FI and CO_2_ emission			
Author's	Region/Countries	Key Variables used and Methods	Findings
[47]	42 OBRI Countries (2007–2019)	ATMs and Debit Cards (2SLS and GMM)	FI rises the CO_2_ emissions
[18]	18 Asian Countires (2004–2019)	BOCB, ATMs, outstanding deposit and loan with commercial banks (Cup-FM and Cup-BC)	FI negative connection with CO_2_ emissions
[48]	China (Province Level)	Peking University Digital FI Index (quantile regression. Mechanism analysis, heterogeneous analysis)	FI negative impact on corban emissions
[20]	284 prefecture-level cities in China	digital FI index (Spatial metrological, Benchmark regression and Robustness test)	FI positive with local cities but negative with neighboring cities.
[49]	31 Asian Countries (2004–2014)	BOCB, ATMs, outstanding deposit and loan with commercial banks (pooled Regression)	FI positive impact on CO_2_ emissions
[50]	BRICS countries (2004–2018)	BOCB, ATMs, outstanding deposit and loan with commercial banks (% of GDP) (Cup-FM and Cup-BC)	FI positive impact on CO_2_ emissions
[16]	APEC countries (2004–2018)	ATMs, BOCB, deposits, and loan (AMG, FMOLS and DOLS)	FI reducing CO_2_ emissions
[40]	E7 countries (from 2004 to 2016)	BOCB, ATMs, outstanding deposit and loan with commercial banks (% of GDP) (Panel quantile regression)	FI positive and negative impact on CO_2_ emissions
**INSQ and CO**_**2**_**emissions**			
[36]	Pakistan	12 INSQ indicator	INSQ has positive impact on CO_2_ emissions
[37]	12 countries	Civil liberty (CL), political right (PR)	INSQ has positive impact on CO_2_ emissions
[51]	African selected countries	Government effectiveness and Regulatory quality	CO_2_ emissions reducing be government effectiveness and regulatory quality
[52]	Forty-nine African nations (1980–2011)	Legal system of institutional quality, Foreign direct investment (GMM)	Strong governance and good INSQ, FDI favorable for the environment

### 2.2 Research gaps in the existing literature

The effectiveness of the FI and INSQ in reducing carbon emissions remains a subject of debate, with mixed empirical evidence. While some studies have demonstrated significant emission reductions resulting from FI and INSQ, others have highlighted their limited impact. Overall, the literature exhibits no consensus on the role of FI and INSQ in reducing carbon emissions, suggesting that further studies are needed. Addressing these mixed findings is crucial, because inconsistent results create uncertainty for policymakers and stakeholders who rely on evidence-based strategies to combat climate change.

This research gap motivated us to explore further dimensions of FI—particularly focusing on ATMs, BOCB, and INSQ—and their relationship with EQ. Investigating the separate impacts of ATMs BOCB and INSQ on EQ will shed light on their specific contributions to emissions in SAARC regions. Although previous studies have examined these factors in isolation, their collective and individual influences on EMS remains underexplored. This study aims to bridge this gap by using the dynamic GMM approach and MM-QR static model to investigate how FI, through BOCB and ATMs, along with INSQ, affects carbon emissions. As proposed in Ref. [53], this methodological approach captures the heterogeneous distributional characteristics of countries across various quantiles of environmental sustainability. Notably, this innovative approach has not been applied in previous studies within the context of SAARC, which also differentiates this study from others.

## 3 Materials and methods

### 3.1 Data and source

This study investigates the impact of FI and INSQ on CO_2_ emissions in SAARC economies, utilizing data from 2004 to 2022. The sample includes seven SAARC countries: Pakistan, Sri Lanka, Bangladesh, Bhutan, Nepal, Maldives, and India. Afghanistan was excluded because of insufficient data on FI and FDI. FI and INSQ were used as predictor variables, whereas CO_2_ emissions were treated as dependent variables. The control variables included FDI, FD, PG, and GI. These variables were selected based on prior studies, such as those by Refs. [16,18,22,26,36,50,51]. Data on CO_2_ emissions, FDI, PG, and FD were sourced from the World Development Indicators, while FI data were obtained from the International Monetary Fund. INSQ data were gathered from the World Governance Indicators and GI data were collected from the KOF Swiss Economic Institute. Table 2 details the measurements of the variables and their respective data sources.

**Table 2:** Description and source of the variables.

Variables	Abbri	Description	Source
Dependent variable			
Carbon Emission	CO2	Metric tons per capita CO_2_ emissions	WDI
Independent variables			
Financial Inclusion	FI	[BOCB]; No. of branches of commercial banks $\left[ATM^{\prime }s\right]$; No. of Automated teller Machines	IMF
Institutional Quality	INSQ	Political stability, Control of Corruption, Rule of Law, Government Effectiveness, Voice & Accountability, and Regulatory Quality	WGI
Control variables			
Forgien Direct Investment	FDI	FDI net inflows with the percentage of GDP	WDI
Population Growth	PG	Annual population growth	WDI
Financial Development	FD	Domestic credit given by banks to private sector (% of GDP)	WDI
Globolization Index	GI	It measures overall dimensions of economic, social and political activities	KOF

### 3.2 Econometric model

The functional form of the empirical model used to investigate the impact of FI and INSQ on CO_2_ emissions is presented as follows:

(1) $${CO}_{2it}=f\left({FI}_{it},{INSQ}_{it},{FDI}_{it},{PG}_{it}{,FD}_{it}{,GI}_{it}\right)$$

Where CO_2_ represents carbon dioxide emissions, FI denotes financial inclusion, INSQ represents an institutional quality, PG denotes population growth, FD stands for financial development, GI represents globalization index, and FDI represents foreign direct investment. The subscripts " i" and " t" refer to nations and time periods, respectively. To empirically test the relationships among FI, INSQ, and CO_2_ emissions, the functional form was transformed into the following econometric specification:

(2) $${CO}_{2it}=\alpha_{it}+\beta_{1}{BOCB}_{it}+\beta_{2}{ATM^{\prime }s}_{it}+\beta_{3}{INSQ}_{it}+\beta_{4}{FDI}_{it}+\beta_{5}{PG}_{it}+\beta_{6}{FD}_{it}+\beta_{7}{GI}_{it}+\varepsilon_{it}$$

Where α represents the slope coefficient, β1, β2, and β3 are the parameters of the independent variables, and β4-7 denotes the coefficients of the control variables. BOCB represents the number of bank branches (a proxy for FI), and ATM represents the number of ATMs (another proxy for FI). The composite error term is denoted by $\varepsilon_{it}$.

### 3.3 Data analysis method

**Fig. 2:**
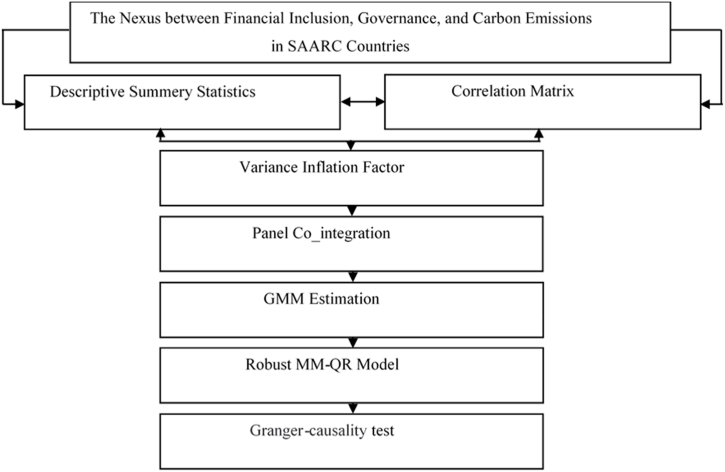
Flow chart of analysis strategy Fig. 2.

The collected data were processed and analyzed using Stata software. Various statistical techniques were employed to achieve the study's objectives, including descriptive statistics, correlation analysis, variance inflation factors (VIF), cross-sectional dependence (CD) tests, panel co-integration tests, and a GMM approach. To ensure the robustness of the results, we employed moment method quantile regression (MM-QR), a static model, and a Granger causality test. Fig. 2 provides a comprehensive overview of the methodological framework used in this study.

The GMM approach was selected to test the research hypotheses because of its several advantages in analyzing dynamic panel data. First, it addresses endogeneity issues by using internal instruments, resulting in more reliable estimates. Second, it is more efficient for correcting heteroskedasticity and serial correlation in error terms, providing more accurate standard errors. Finally, the GMM approach pools equations in levels and first differences, allowing it to capture both short- and long-run relationships, making it particularly suitable for cases with limited sample sizes and potential non-linear interactions among variables. Following Equation (2), the econometric model is further expanded into three dynamic GMM model equations as follows:

(3) $${CO}_{2it}=\alpha_{0}+\beta_{1}{\left(CO_{2}\right)}_{it-1}+\beta_{2}{\left(BOCB\right)}_{it}+\beta_{3}{\left(INSQ\right)}_{it}+\beta_{4}{\left(Control\;Factors\right)}_{it}+\mu_{i}+\varepsilon_{it}$$

(4) $${CO}_{2it}=\alpha_{0}+\beta_{1}{\left(CO_{2}\right)}_{it-1}+\beta_{2}{\left(ATM^{'}s\right)}_{it}+\beta_{3}{\left(INSQ\right)}_{it}+\beta_{4}{\left(Control\;Factors\right)}_{it}+\mu_{i}+\varepsilon_{it}$$

(5) $${CO}_{2it}=\alpha_{0}+\beta_{1}{\left(CO_{2}\right)}_{it-1}+\beta_{2}{\left(BOCB\right)}_{it}+\beta_{3}{\left(ATM^{'}s\right)}_{it}+\beta_{4}{\left(INSQ\right)}_{it}+\beta_{5}{\left(Control\;Factors\right)}_{it}+\mu_{i}+\varepsilon_{it}$$

To validate the results of the dynamic analysis further, the dynamic GMM approach was applied to estimate Equations (3), (4), (5). For comparison purposes, a robust static analysis was conducted to assess the impact of the independent variables on carbon emissions. In this static analysis, ${CO2}_{it-1}$ was excluded from the models and the “Driscoll-Kraay standard error technique” [54] was employed to estimate the parameters. Additionally, MM-QR, as outlined by Ref. [53], was used to ensure the robustness of the static analysis results. To provide further insight into the causal relationships between FI, its components, and CO_2_ emissions, the panel Granger non-causality test developed by Ref. [55] was applied. This test, which accounts for CD, tests the null hypothesis of no causal relationship with the alternative causal relationship.

## 4 Empirical results and discussion

### 4.1 Descriptive statistics

Table 3 presents the descriptive statistics for all the variables included in the analysis, which provides an overview of the data distribution, central tendency, and variability, offering valuable insights into the key features of the dataset. The average values of CO_2_, BOCB, ATMs, INSQ, FDI, PG, FD, and GI are 1.10, 7.52, 6.78, 0.04, 2.25, 1.43, 40.07, and 49.33, respectively. The standard deviations for CO_2_, BOCB, ATMs, INSQ, FDI, PG, FD, and GI are 0.86, 2.68, 2.85, 1.00, 3.27, 0.89, 16.53, and 8.60, respectively. The mean, standard deviation, and minimum and maximum values of these variables were consistent with the patterns observed in similar studies [56]. Overall, the descriptive statistics indicate that the data are normally distributed, providing a solid foundation for subsequent empirical analyses.

**Table 3:** Descriptive statistics.

Variables	Mean	Std. dev	Min	Max
CO2	1.104067	0.8602461	0.0986501	3.963267
BOCB	7.521411	2.680399	2.772589	11.92748
$ATM^{\prime }s$	6.776958	2.848066	0.6931472	12.47179
INSQ	0.0350708	0.9988815	−1.00346	2.37891
FDI	2.248283	3.272885	−0.6388063	16.78347
PG	1.427295	0.8852526	0.1127726	4.422534
FD	40.06582	16.53157	13.80499	103.5262
GI	49.33083	8.595913	27	63

### 4.2 Correlation matrix

Table 4 presents the correlations between the variables, offering preliminary insights into the relationships explored in this study. FI, represented by ATM's (r = 0.1391; p < 0.05) and BOCB (r = 0.436; p < 0.05), shows a significant positive correlation with CO_2_, suggesting that higher FI is associated with increasing CO_2_ emissions. INSQ also exhibits a significant positive correlation with CO_2_, with a coefficient of 0.462 (p < 0.05), indicating that stronger INSQ, possibly reflecting a more robust regulatory frameworks, is linked with higher carbon emissions.

**Table 4:** Results of Correlations matrix.

Variables	CO_2_	BOCB	ATM's	INSQ	FDI	PG	FD	GI
CO2	1.00							
BOCB	0.436∗ 0.000	1.00						
$ATM^{\prime }s$	0.1391∗ 0.000	0.852∗ 0.000	1.00					
INSQ	0.462∗ 0.000	−0.737∗ 0.000	−0.585∗ 0.000	1.00				
FDI	0.721∗ 0.000	−0.498∗ 0.000	−0.285∗ 0.000	0.272∗ 0.000	1.00			
PG	0.629∗ 0.000	−0.352∗ 0.000	−0.182∗ 0.035	0.119 0.170	0.665∗ 0.000	1.00		
FD	0.008 0.822	0.038 0.659	0.179∗ 0.038	0.186∗ 0.031	−0.133 0.125	−0.243∗ 0.004	1.00	
GI	−0.090 0.302	0.719∗ 0.000	0.619∗ 0.000	−0.550∗ 0.000	−0.016 0.848	−0.109 0.208	−0.023 0.791	1.00

Notes: The symbols ∗ denote the levels of significance of values at 5 %.

### 4.3 Multicollinearity test

Table 5 presents the results of the VIF test used to assess multicollinearity among the independent variables. The VIF values for all variables are within the acceptable range, indicating no significant multicollinearity concerns.

**Table 5:** Results of multicollinearity test – Variance Inflation Factor (VIF).

Variables	VIF	1/VIF
BOCB	2.77	0.201928
$ATM^{\prime }s$	2.93	0.212662
INSQ	2.35	0.201594
FDI	2.32	0.201442
PG	2.55	0.392269
FD	2.00	0.500609
GI	1.30	0.770116
Mean	2.38	–

### 4.4 Cross-sectional dependence (CD) test

We conducted CD analysis to assess the dependency characteristics of the study variables. Following the recommendations of [57], we employed second-generation panel unit root testing methods, which are more suitable than first-generation tests for handling high cross-sectional dependency. Testing for residual CD is crucial before proceeding with econometric analyses. The CD test, as introduced by Ref. [58], calculates the average pairwise correlation coefficients of ordinary least square residuals from augmented Dickey-Fuller regressions applied to each panel variable. Under the null hypothesis of CD, the CD test statistic follows a two-tailed standard normal distribution. Table 6 demonstrates that all variables, except for FDI, exhibit significant CD, rejecting the null hypothesis at the 1 % significance level for most variables. This CD is considered by our GMM estimator.

**Table 6:** Results of cross-sectional dependence test.

Variables	CD Test	P-Value
CO2	16.50∗∗∗	0.000
BOCB	19.02∗∗∗	0.000
$ATM^{\prime }s$	18.44∗∗∗	0.000
INSQ	8.68∗∗∗	0.000
FDI	0.43	0.670
PG	2.37∗∗	0.018
FD	2.36∗∗	0.018
GI	11.49∗∗∗	0.000

Notes: The symbols ∗∗, and ∗∗∗ denote the levels of significance of values at 5 %, and 1 % respectively.

### 4.5 Panel co-integration test

Table 7 presents the results of the co-integration tests, which assess whether the nonstationary time series exhibits stable long-term relationships. The Westerlund variance ratio and Pedroni co-integration tests indicate that the panels are cointegrated, suggesting that the variables have a stable, long-term relationship.

**Table 7:** Results of panel co-integration test.

Tests for Co-integration	Time trend: Included	Co-integration vector and AR parameter: Panel Specific	Decision
**Westerlund Variance Ratio**	Yes	1.513∗	Panels are co-integrated
**Pedroni co-integration test**	Kernel: Bartlett with (2.00) Newey west Lags and augmented lags: 1(AIC)		
Modified Phillips–Perron t	Yes	4.103∗∗∗	Panels are co-integrated
Phillips–Perron t	Yes	−3.412∗∗∗	
Augmented Dickey–Fuller t	Yes	−3.216∗∗∗	

Notes: The symbols ∗, ∗∗, and ∗∗∗ denote the levels of significance of values at 1 %, 5 %, and 10 %, respectively.

### 4.6 Regression analyses

We performed a dynamic GMM regression analysis to formally test the hypotheses, and the results are presented in Table 8, where columns (1), (2), and (3) display the estimates for Eq. (3), Eq. (4), and Eq. (5), respectively. Prior to performing the baseline test, we assess the validity of the system GMM estimator. The estimator is valid if the instruments are exogenous (verified by the Hansen test of over-identifying restrictions) and if there is no second-order autocorrelation in the errors (as confirmed by the Arellano-Bond AR2 test). In our analysis, the AR1 statistics are negative and statistically significant (p-value <0.05), whereas the AR2 statistics are not significant (p-value >0.10), indicating that the error term ($\varepsilon_{it}$) is not serially correlated, and the instruments are exogenous. The Hansen test p-value exceeding 0.10 further confirms the validity of the instruments and the GMM estimator. Thus, the dynamic GMM estimator was suitable for this analysis. The coefficient of Lag1CO_2_ is positive and significant, suggesting that FI and INSQ in the previous year positively influence carbon emissions in the current year. This implies that a dynamic approach is more appropriate for investigating the effects of FI and INSQ on CO_2_ emissions than static ones.

**Table 8:** Dynamic GMM estimation.

Variables	(1)	(2)	(3)
Lag1CO2	0.112∗∗∗ (0.068)	0.279∗∗∗ (0.151)	0.213∗∗∗ (0.131)
BOCB	0.017∗∗∗ (0.121)	–	0.793∗∗∗ (0.503)
$ATM^{\prime }s$	–	0.181∗∗∗ (0.101)	0.079∗∗ (0.018)
INSQ	0.130∗∗ (0.017)	0.074∗∗∗ (0.048)	0.213∗∗∗ (0.014)
FDI	0.023∗∗∗ (0.010)	0.085∗∗ (0.138)	0.200∗∗∗ (0.176)
PG	0.031 (0.001)	0.078∗ (0.015)	0.092∗∗ (0.003)
FD	0.171∗∗ (0.025)	0.053∗∗∗ (0.025)	0.794∗∗∗ (0.323)
GI	−0.014∗∗∗ (0.010)	−0.083∗∗∗ (0.201)	−0.068∗∗∗ (0.471)
Constant	2.394∗∗∗ (0.302)	2.786∗∗∗ (0.472)	1.638∗∗∗ (0.532)
**Post-Analysis**			
AR1 (P-value)	−2.463 (0.029)	−2.388 (0.005)	−3.748 (0.001)
AR2 (P-value)	0.655 (0.320)	0.389 (0.250)	0.529 (0.697)
Sargan test value	273.8	239.2	482.2
Hansen test value	59.23	57.43	49.32
No of Instruments	86	85	85
No of observation	119	119	119

Notes: The symbols ∗, ∗∗, and ∗∗∗ denote the levels of significance of values at 1 %, 5 %, and 10 %, respectively, and brackets represent the standard error values of the models.

Turning to the baseline variables, the results in columns (1) and (3) indicate that the coefficient of BOCB is positive and statistically significant. A 1 % increase in the number of bank branches is associated with 0.017 % and 0.793 % increases in CO_2_ emissions, respectively. These findings imply that the expansion of banking infrastructure contributes to economic growth, which in turn drives higher energy demand and increased carbon emissions [59]. Although FI fosters access to credit and supports economic activity, it can also have negative environmental consequences by increasing energy consumption through heightened production and consumption. These results align with [60].

In columns (2) and (3), the positive and statistically significant coefficients of the number of ATMs suggest a similar relationship between FI and carbon emissions. A 1 % increase in the number of ATMs corresponds to 0.181 % and 0.079 % increase in emissions, respectively. These results are consistent with [47], who find that ATMs to higher carbon emissions in selected OBRI economies. While FI enhances access to financial services, benefiting both consumers and institutions, it enables individuals to obtain the capital needed for essential needs, such as food, shelter, healthcare, education, and consumer goods. On the supply side, growth of the financial sector, resulting from increased inclusiveness, creates a more dynamic and stable financial system by introducing new credit facilities. This allows banks and financial institutions to offer a wider range of products and services to both existing and new customers, increase income streams for these institutions, and drive overall economic growth [61]. However, this surge in financial activities, including the use of ATMs, online payments, ATM withdrawals, and mobile money applications, leads to higher energy consumption and resource use, thereby increasing carbon emissions. It is well established that social and economic activities driven by individuals are among the major contributors to CO_2_ emissions. With greater financial inclusiveness, people gain easier access to financial services and feel more empowered to carry out their social and economic roles, which indirectly contributes to CO_2_ emissions [19]. Furthermore, FI plays a crucial role in FD, stimulating production and industrialization, which are key sources of CO_2_ emissions [47]. The overall positive impact of FI on CO_2_ emissions is consistent with [7] and these findings support H_1_.

The positive and significant INSQ coefficients across all three columns indicate that improvements in INSQ are associated with higher CO_2_ emissions. These findings support the notion that improvements in INSQ might coincide with increased economic activity, which in turn could lead to greater carbon emissions, especially in developing countries. These findings are consistent with those of [62]. Higher INSQ may offer greater political and civil freedom, leading to increased industrial activities and, consequently, higher emissions. This may reflect a lag in environmental policy implementation, where governments prioritize industrial development over pollution control. As institutions improve, particularly in emerging economies, their focus might be more on economic expansion and infrastructure development, which increases energy consumption and emissions. Additionally, in developing countries, the implementation of policies related to environmental protection often delays economic goals, and institutional advancements may prioritize immediate economic needs over long-term sustainability. This finding supports hypothesis H_2_.

Regarding the control variables, the coefficients for FDI and FD are both positive and significant, suggesting that increased FDI and financial development contribute to higher CO_2_ emissions in SAARC countries. FDI-driven industrialization and infrastructure development often lead to increased energy use and pollution. This highlights the environmental costs of attracting foreign investment and expanding financial markets in regions with weak environmental regulations. Similarly, PG also shows a positive relationship with CO_2_ emissions, as growing populations increase the demand for energy, transportation, housing, and industrial output, all of which contribute to higher emissions. In contrast, the GI shows a significant negative correlation with CO_2_ emissions, implying that increased globalization in these countries may help reduce emissions by facilitating the exchange of eco-friendly technologies and encouraging local industries to adopt more sustainable practices. This finding supports the idea that integration into global markets can enhance environmental outcomes by promoting cleaner production methods and higher standards.

### 4.7 Robustness tests

We conducted additional robustness checks to further validate our findings on the effects of FI and INSQ on CO_2_ emissions. The robust results from the static analysis, presented in Table 9, Table 10, Table 11, reaffirm the positive relationship between BOCB, ATMs, and INSQ with CO_2_ emissions. However, the magnitude of these effects varies across the selected quantiles, suggesting that the influence of FI and INSQ differs depending on economic context. Given that both the dynamic and static models consistently show a positive relationship between FI, INSQ, and carbon emissions, it is crucial to explore the causal direction of these relationships. To address this, we employed the panel Granger non-causality test developed by Ref. [55]. The results, shown in Table 12, reveal bidirectional causality from ATM to carbon emissions, unidirectional causality between BOCB and CO_2_, and bidirectional causal effects from both ATMs and INSQ on CO_2_ emissions. These findings highlight the significant roles of FI and INSQ in driving carbon emissions. Focusing on these relationships is key to achieving carbon reduction goals. To make meaningful progress in reducing emissions and enhancing EQ, SAARC nations should prioritize policies that address the environmental impact of FI and INSQ while advancing their sustainability targets.

**Table 9:** Regression models. (3), (4) and (5) without including ${CO2}_{it-1}$ as explanatory variable; CO_2_ is the dependent variable.

Variables	Driscoll-Kraay standard errors			Lower Quantile Levels								
				(Q=.1)			(Q=.2)			(Q=.3)		
	(1)	(2)	(3)	(1)	(2)	(3)	(1)	(2)	(3)	(1)	(2)	(3)
BOCB	0.012∗∗∗ (0.040)		0.197∗∗∗ (0.044)	0.012∗∗∗ (0.030)		0.111∗∗∗ (0.065)	0.011∗∗∗ (0.026)		0.135∗∗∗ (0.055)	0.013∗∗∗ (0.031)		0.160∗∗∗ (0.048)
$ATM^{\prime }s$		0.063∗∗ (0.022)	0.014∗∗∗ (0.028)		0.053∗∗∗ (0.012)	0.175∗∗∗ (0.017)		0.057∗∗∗ (0.010)	0.169∗∗∗ (0.016)		0.059∗∗∗ (0.009)	0.165∗∗∗ (0.016)
INSQ	0.426∗∗∗ (0.086)	0.022∗∗∗ (0.075)	0.389∗∗∗ (0.056)	0.339∗∗∗ (0.057)	0.479∗∗∗ (0.060)	0.363∗∗∗ (0.035)	0.364∗∗∗ (0.052)	0.485∗∗∗ (0.052)	0.369∗∗∗ (0.033)	0.381∗∗∗ (0.049)	0.489∗∗∗ (0.048)	0.373∗∗∗ (0.032)
FDI	0.127∗∗∗ (0.019)	0.144∗∗∗ (0.015)	0.096∗∗∗ (0.016)	0.121∗∗∗ (0.021)	0.128∗∗∗ (0.021)	0.068∗∗∗ (0.014)	0.123∗∗∗ (0.018)	0.133∗∗∗ (0.018)	0.075∗∗∗ (0.013)	0.124∗∗∗ (0.017)	0.137∗∗∗ (0.017)	0.078∗∗∗ (0.013)
PG	0.284∗∗∗ (0.066)	0.258∗∗∗ (0.062)	0.245∗∗∗ (0.034)	0.300∗∗∗ (0.059)	0.227∗∗∗ (0.071)	0.209∗∗∗ (0.036)	0.295∗∗∗ (0.053)	0.237∗∗∗ (0.062)	0.217∗∗∗ (0.034)	0.292∗∗∗ (0.049)	0.243∗∗∗ (0.057)	0.222∗∗∗ (0.34)
FD	0.002∗∗ (0.003)	0.000 (0.003)	0.001∗∗ (0.002)	0.002 (0.000)	0.001∗∗∗ (0.000)	0.003∗∗ (0.001)	0.002∗∗ (0.001)	0.005∗∗∗ (0.002)	0.004∗∗ (0.001)	0.003∗∗∗ (0.001)	0.001∗∗ (0.000)	0.004∗∗∗ (0.001)
GI	−0.043∗∗∗ (0.002)	0.031∗∗∗ (0.002)	−0.051∗∗∗ (0.005)	−0.042∗∗∗ (0.007)	0.048∗∗∗ (0.005)	0.064∗∗ (0.005)	−0.042∗∗∗ (0.006)	0.043∗∗∗ (0.004)	0.061∗∗∗ (0.004)	−0.042∗∗∗ (0.006)	−0.039∗∗∗ (0.004)	−0.059∗∗∗ (0.004)
Constant	−1.739∗∗∗ (0.186)	−1.581∗∗∗ (0.192)	−1.445∗∗∗ (0.183)	−2.123∗∗∗ (0.027)	−2.554∗∗∗ (0.353)	−1.801∗∗∗ (0.170)	−2.012∗∗∗ (0.242)	−2.248∗∗∗ (0.298)	−1.721∗∗∗ (0.160)	−1.938∗∗∗ (0.230)	−2.037∗∗∗ (0.275)	−1.672∗∗∗ (0.159)
Obs.	133	133	133	133	133	133	133	133	133	133	133	133

Notes: The symbols ∗, ∗∗, and ∗∗∗ denote the levels of significance of values at 1 %, 5 %, and 10 %, respectively, and brackets represent the standard error values of the models.

**Table 10:** Regression models. (3), (4) and (5) without including ${CO2}_{it-1}$ as explanatory variable; CO_2_ is the dependent variable.

Variables	Median Quantile Levels								
	(Q=.4)			(Q=.5)			(Q=.6)		
	(1)	(2)	(3)	(1)	(2)	(3)	(1)	(2)	(3)
BOCB	0.010∗∗∗ (0.024)		0.190∗∗∗ (0.041)	0.013∗∗∗ (0.025)		0.213∗∗∗ (0.037)	0.013∗∗∗ (0.023)		0.230∗∗∗ (0.034)
$ATM^{\prime }s$		0.061∗∗∗ (0.009)	0.160∗∗∗ (0.016)		0.063∗∗∗ (0.009)	0.154∗∗∗ (0.018)		0.065∗∗∗ (0.009)	0.145∗∗∗ (0.020)
INSQ	0.409∗∗∗ (0.046)	0.495∗∗∗ (0.045)	0.378∗∗∗ (0.033)	0.423∗∗∗ (0.046)	0.498∗∗∗ (0.044)	0.384∗∗∗ (0.035)	0.442∗∗∗ (0.048)	0.501∗∗∗ (0.045)	0.392∗∗∗ (0.039)
FDI	0.126∗∗∗ (0.016)	0.141∗∗∗ (0.015)	0.084∗∗∗ (0.013)	0.127∗∗∗ (0.016)	0.144∗∗∗ (0.015)	0.090∗∗∗ (0.014)	0.128∗∗∗ (0.017)	0.146∗∗∗ (0.016)	0.098∗∗∗ (0.016)
PG	0.287∗∗∗ (2.59)	0.252∗∗∗ (0.053)	0.229∗∗∗ (0.034)	0.285∗∗∗ (0.047)	0.258∗∗∗ (0.053)	0.237∗∗∗ (0.037)	0.281∗∗∗ (0.048)	0.261∗∗∗ (0.053)	0.248∗∗∗ (0.041)
FD	0.002∗∗∗ (0.001)	0.000∗∗ (0.002)	0.005∗∗∗ (0.003)	0.002∗∗∗ (0.017)	0.001∗∗ (0.002)	0.006∗∗∗ (0.003)	0.003∗∗∗ (0.019)	0.000∗∗ (0.002)	0.007∗∗∗ (0.003)
GI	−0.043∗∗∗ (0.005)	−0.034 (0.004)	−0.057∗∗ (0.004)	−0.040∗∗∗ (0.005)	−0.031∗∗∗ (0.004)	−0.054∗∗∗ (0.005)	−0.043∗∗∗ (0.006)	−0.029∗∗∗ (0.004)	−0.049∗∗∗ (0.005)
Constant	−1.810∗∗∗ (0.217)	−1.767∗∗∗ (0.256)	−1.601∗∗∗ (0.163)	−1.749∗∗∗ (0.217)	−1.580∗∗∗ (0.254)	−1.520∗∗∗ (0.174)	−1.667∗∗∗ (0.223)	−1.460∗∗∗ (0.262)	−1.411∗∗∗ (0.196)
Obs.	133	133	133	133	133	133	133	133	133

Notes: The symbols ∗, ∗∗, and ∗∗∗ denote the levels of significance of values at 1 %, 5 %, and 10 %, respectively and brackets consist standard error value of the model.

**Table 11:** Regression models. (3), (4) and (5) without including ${CO2}_{it-1}$ as explanatory variable; CO_2_ is the dependent variable.

Variables	Upper Quantile Levels								
	(Q=.7)			(Q=.8)			(Q=.9)		
	(1)	(2)	(3)	(1)	(2)	(3)	(1)	(2)	(3)
BOCB	0.013∗∗∗ (0.026)		0.245∗∗∗ (0.033)	0.012∗∗∗ (0.029)		0.256∗∗∗ (0.033)	0.019∗∗∗ (0.034)		0.273∗∗∗ (0.035)
$ATM^{\prime }s$		0.067∗∗∗ (0.009)	0.134∗∗∗ (0.024)		0.071∗∗∗ (0.011)	0.125∗∗∗ (0.027)		0.074∗∗∗ (0.013)	0.117∗∗∗ (0.032)
INSQ	0.463∗∗∗ (0.051)	0.506∗∗∗ (0.048)	0.403∗∗∗ (0.047)	0.491∗∗∗ (0.058)	0.513∗∗∗ (0.056)	0.411∗∗∗ (0.055)	0.523∗∗∗ (0.066)	0.520∗∗∗ (0.065)	0.420∗∗∗ (0.063)
FDI	0.130∗∗∗ (0.018)	0.151∗∗∗ (0.017)	0.109∗∗∗ (0.019)	0.131∗∗∗ (0.020)	0.157∗∗∗ (0.019)	0.118∗∗∗ (0.022)	0.134∗∗∗ (0.024)	0.162∗∗∗ (0.022)	0.127∗∗∗ (0.026)
PG	0.278∗∗∗ (0.052)	0.269∗∗∗ (0.057)	0.263∗∗∗ (0.049)	0.273∗∗∗ (0.058)	0.280∗∗∗ (0.066)	0.274∗∗∗ (0.057)	0.267∗∗∗ (0.068)	0.290∗∗∗ (0.077)	0.286∗∗∗ (0.066)
FD	0.002∗∗ (0.000)	0.000∗∗ (0.002)	0.007∗∗∗ (0.004)	0.002∗∗∗ (0.001)	.0.001∗∗∗ (0.000)	0.007∗∗ (0.005)	0.003∗∗ (0.000)	0.001∗∗∗ (0.003)	0.008∗∗∗ (0.005)
GI	−0.043∗∗∗ (0.006)	−0.025∗∗∗ (0.004)	−0.044∗∗ (0.006)	−0.043∗∗∗ (0.007)	−0.018∗∗∗ (0.005)	−0.039∗∗∗ (0.007)	−0.043∗∗∗ (0.008)	−0.013∗∗∗ (0.005)	−0.035∗∗∗ (0.009)
Constant	−1.575∗∗∗ (0.239)	−1.198∗∗∗ (0.284)	−1.269∗∗∗ (0.232)	−1.453∗∗∗ (0.269)	−0.846∗∗∗ (0.316)	−1.153∗∗∗ (0.267)	−1.313∗∗∗ (0.313)	−0.535∗∗∗ (0.356)	−1.038∗∗∗ (0.311)
Obs.	133	133	133	133	133	133	133	133	133

Notes: The symbols ∗, ∗∗, and ∗∗∗ denote the levels of significance of values at 1 %, 5 %, and 10 %, respectively and brackets consist standard error value of the model.

**Table 12:** Granger-causality test results (Dumitrescu & Hurlin, 2012).

Direction/Route	W-Statistics	Z-bar Statistics	P-Value
CO_2_ → BOCB	1.3311	0.6195	0.535
BOCB → CO_2_	9.4713∗∗∗	15.8484	0.000
CO_2_ → ATM's	2.0745∗∗	2.0101	0.044
ATM's → CO_2_	15.2618∗∗∗	26.6813	0.000
CO_2_ → INSQ	6.9304∗∗∗	11.0947	0.000
INSQ→ CO_2_	5.1311∗∗∗	7.7286	0.000

Notes: The symbols ∗, ∗∗, and ∗∗∗ denote the levels of significance of values at 1 %, 5 %, and 10 %, respectively.

## 5 Conclusion and implications

This study examines the impact of FI and INSQ on CO_2_ emissions in the SAARC economies, analyzing data from 2004 to 2022. The research hypotheses were tested using a GMM approach, which provides dynamic insights into the relationships between the variables. To validate the results further, MM-QR, static model, and Granger causality tests were employed.

The dynamic GMM results reveal that both FI indicators, BOCB and ATMs, are positively associated with CO_2_ emissions. An increase in the number of bank branches correlates with higher CO_2_ emissions, suggesting that the expansion of banking infrastructure contributes to greater financial activity, and consequently, increased energy consumption, leading to greater carbon emissions. Similarly, the number of ATMs also has a positive impact on emissions, indicating that greater financial inclusiveness, while beneficial for economic development, contributes to a higher carbon footprint. These finding suggest that the expansion of financial infrastructure drives economic growth, but at the cost of environmental degradation. Increased access to financial services stimulates higher energy consumption and production activities, leading to greater carbon emissions. Moreover, INSQ has a significant positive impact on CO_2_ emissions, with better INSQ—reflecting robust regulatory frameworks and governance—also associated with higher emissions. This supports the notion that improvements in INSQ may coincide with increased economic activity, particularly in developing countries, leading to greater carbon emissions. Control variables such as FDI, FD, and PG further compound the issue as they are positively and significantly affecting CO_2_ emissions, highlighting the environmental trade-offs of economic development in SAARC nations. However, GI appears to mitigate emissions, suggesting that greater integration into global markets may encourage the adoption of cleaner technologies and practices.

Robustness tests further support these results, showing that the effects of FI and INSQ on CO_2_ emissions vary across economic contexts, as indicated by quantile analyses. The panel Granger non-causality test confirms a mixed causal relationship between FI and CO_2_ emissions, such as the unidirectional causality between bank branches and CO_2_. The findings also demonstrate the bidirectional causal effect of ATMs and INSQ on carbon emissions, reinforcing the argument that the expansion of financial services and improvements in INSQ drive higher emissions in the SAARC region.

Overall, this study concludes that FI and INSQ contribute to higher carbon emissions in the SAARC countries. Policymakers must strike a balance between fostering economic development and ensuring sustainable environmental outcomes. Future strategies should focus on integrating environmentally friendly technologies and policies to mitigate the negative effects of FI and institutional growth on carbon emissions.

### 5.1 Practical implications

The findings of this study have significant practical implications for policymakers and stakeholders in the SAARC countries. The positive relationship between FI, INSQ, and CO_2_ emissions underscores the need for a balanced approach to economic and environmental policy. Policymakers should recognize that, while expanding financial infrastructure and improving INSQ are essential for economic development, these advancements can lead to increased carbon emissions. To mitigate these effects, it is crucial to integrate environmentally friendly practices and technologies into the financial and institutional development strategies. Promoting green banking initiatives and incorporating sustainability criteria into financial services can help reduce the environmental impacts of FI. Furthermore, improving institutional frameworks that emphasize environmental regulations and sustainable practices can help align economic growth with environmental goals. Policymakers should also encourage the adoption of clean technologies and energy-efficient practices across sectors. By fostering a more sustainable approach to financial and institutional development, SAARC countries can achieve economic growth while minimizing adverse environmental impacts, contributing to the broader goal of reducing carbon emissions and enhancing the overall EQ.

The positive relationship between FDI, FD, PG, and CO_2_ emissions highlights the environmental trade-offs of economic expansion. Policymakers should attract investment and foster FD while prioritizing environmental sustainability. This can be achieved by encouraging industries to adopt cleaner technologies and promoting policies that balance economic growth with environmental protection. Conversely, the negative impact of GI on CO_2_ emissions offers a potential avenue for reducing carbon footprints. By facilitating the transfer of environmentally friendly technologies and practices, globalization can contribute to sustainable development. Policymakers should leverage global integration to enhance environmental standards and encourage the adoption of green technologies. Overall, this study emphasizes the importance of integrating environmental considerations into economic and financial policies. To achieve sustainable development, SAARC nations must adopt strategies that balance FI and institutional improvements, with effective measures to reduce carbon emissions.

## 6 Limitation and future research direction

This study has several limitations that should be considering in future research. First, the analysis could not include data from all eight SAARC countries because of unavailability, which may limit the comprehensiveness of the findings across the entire region. Second, reliance on panel data aggregated at the SAARC level restricts the ability to derive nuanced, country-specific estimates. Although countries within SAARC share several common features, such as income levels, institutional development, culture, and geographic proximity, they also exhibit significant differences. For instance, India's economic advancement surpasses that of its regional counterparts, and conflicts among neighboring states may yield divergent explanations. To address these limitations, future research should focus on conducting more granular analyses within individual SAARC countries to uncover specific national dynamics. Comparative studies between SAARC nations and similar low-income or developing economies could also offer deeper insights into how FI and INSQ affect CO_2_ emissions in diverse contexts.

## CRediT authorship contribution statement

**Jafir Mehmood:** Writing – original draft, Methodology, Formal analysis, Conceptualization. **Yang Jinghan:** Writing – review & editing, Software, Methodology, Investigation, Formal analysis. **Jing Wang:** Writing – review & editing, Conceptualization, Validation, Data curation, Methodology, Supervision, Funding acquisition. **Maqsood Ahmad:** Writing – review & editing, Validation, Resources, Funding acquisition, Data curation, Conceptualization.

## Data availability

The requested data could be obtained by making a formal request to the corresponding author.

## Declaration of competing interest

I would like to declare on behalf of my co-author that the work described is original research that has not been published previously, and not under consideration for publication elsewhere, in whole or in part. I confirmed that no conflict of interest exists in the submission of this manuscript, and is approved by all authors for publication in your journal.
